# The synergistic antibacterial activity and mechanism of colistin-oxethazaine combination against gram-negative pathogens

**DOI:** 10.3389/fphar.2024.1363441

**Published:** 2024-03-21

**Authors:** Jie Li, Ning Han, Yangyang Li, Feifei Zhao, Wenguang Xiong, Zhenling Zeng

**Affiliations:** ^1^ Guangdong Provincial Key Laboratory of Veterinary Pharmaceutics Development and Safety Evaluation, College of Veterinary Medicine, South China Agricultural University, Guangzhou, China; ^2^ National Laboratory of Safety Evaluation (Environmental Assessment) of Veterinary Drugs, South China Agricultural University, Guangzhou, China; ^3^ National Risk Assessment Laboratory for Antimicrobial Resistance of Animal Original Bacteria, South China Agricultural University, Guangzhou, China

**Keywords:** antibiotic resistance, drug combination, colistin, oxethazine, gram-negative bacteria

## Abstract

**Background:**

The rapid spread of bacteria with plasmid-mediated resistance to antibiotics poses a serious threat to public health. The search for potential compounds that can increase the antibacterial activity of existing antibiotics is a promising strategy for addressing this issue.

**Methods:**

Synergistic activity of the FDA-approved agent oxethazine combined with colistin was investigated *in vitro* using checkerboard assays and time-kill curves. The synergistic mechanisms of their combination of oxethazine and colistin was explored by fluorescent dye, scanning electron microscopy (SEM) and LC-MS/MS. The synergistic efficacy was evaluated *in vivo* by the *Galleria mellonella* and mouse sepsis models.

**Results:**

In this study, we found that oxethazine could effectively enhance the antibacterial activity of colistin against both *mcr*-positive and -negative pathogens, and mechanistic assays revealed that oxethazine could improve the ability of colistin to destruct bacterial outer membrane and cytoplasmic membrane permeability. In addition, their combination triggered the accumulation of reactive oxygen species causing additional damage to the membrane structure resulting in cell death. Furthermore, oxethazine significantly enhanced the therapeutic efficacy of colistin in two animal models.

**Conclusion:**

These results suggested that oxethazine, as a promising antibiotic adjuvant, can effectively enhance colistin activity, providing a potential strategy for treating multidrug-resistant bacteria.

## 1 Introduction

The discovery and clinical use of antibiotics was one of the highlights of the 20th century, which resulted in an average increase in human life expectancy of 23 years (Katz and Baltz, 2016; Hutchings et al., 2019). However, as a double-edged sword, antimicrobial resistance caused by overuse of antibiotics is growing more serious and the therapeutic alternatives are becoming more difficult due to multidrug-resistant (MDR) bacteria (McEwen and Collignon, 2018; Pariente and PLOS Biology Staff Editors, 2022). Furthermore, the World Health Organization (WHO) considers it to be one of the top 10 public health threats (Pariente and PLOS Biology Staff Editors, 2022), with MDR Gram-negative bacteria being of particular concern (Silvestri and van Saene, 2010). The permeability barrier of outer membrane (OM) of Gram-negative bacteria makes it difficult for the drugs to enter the cell and reach the site of action (Pagès et al., 2008; MacNair and Brown, 2020). Despite this, the majority of antibiotics currently in use to treat Gram-negative bacteria are from classes discovered before the 1970s (Lewis, 2013; Cook and Wright, 2022). Only two new antibiotics have been approved in the last 20 years, both against Gram-positive bacteria (MacNair and Brown, 2020). Despite the urgent need for new antibiotics to treat MDR Gram-negative infections, no new Gram-negative antibiotics have been identified since the quinolones (Lewis, 2020; MacNair and Brown, 2020). Therefore, developing new therapeutic strategies to tackle antibiotic resistance in Gram-negative bacteria is critical.

Combination therapies offer a promising strategy to overcome the antibiotic resistance crisis (Bailey et al., 2022). Combination therapy was discovered in the 1950s to combat single-drug resistance (Jayaraman et al., 2010; Kerantzas and Jacobs, 2017), and it has proven to be more effective than monotherapy (Bailey et al., 2022). Moreover, combination therapy is an effective strategy to reduce the time and cost of developing traditional antibiotics (Liu et al., 2021). Colistin has been used as a last resort against MDR Gram-negative pathogens (Paterson and Harris, 2016; Yin et al., 2021). The preventive use of colistin as a food additive led to an increase in colistin resistance rates in the livestock and poultry industry (Sun et al., 2020). The *mcr-1* gene encodes phosphoethanolamine (pEtN) transferase that transfers pEtN to lipid A, resulting in colistin resistance (Liu et al., 2023). Notably, the antibacterial efficacy of colistin has been threatened by the emergence of the mobile drug resistance gene *mcr-1* since 2015 (Liu et al., 2016; Yin et al., 2021). Therefore, it is vital to find colistin adjuvants for treating infections caused by colistin-resistant bacteria.

**FIGURE 1:**
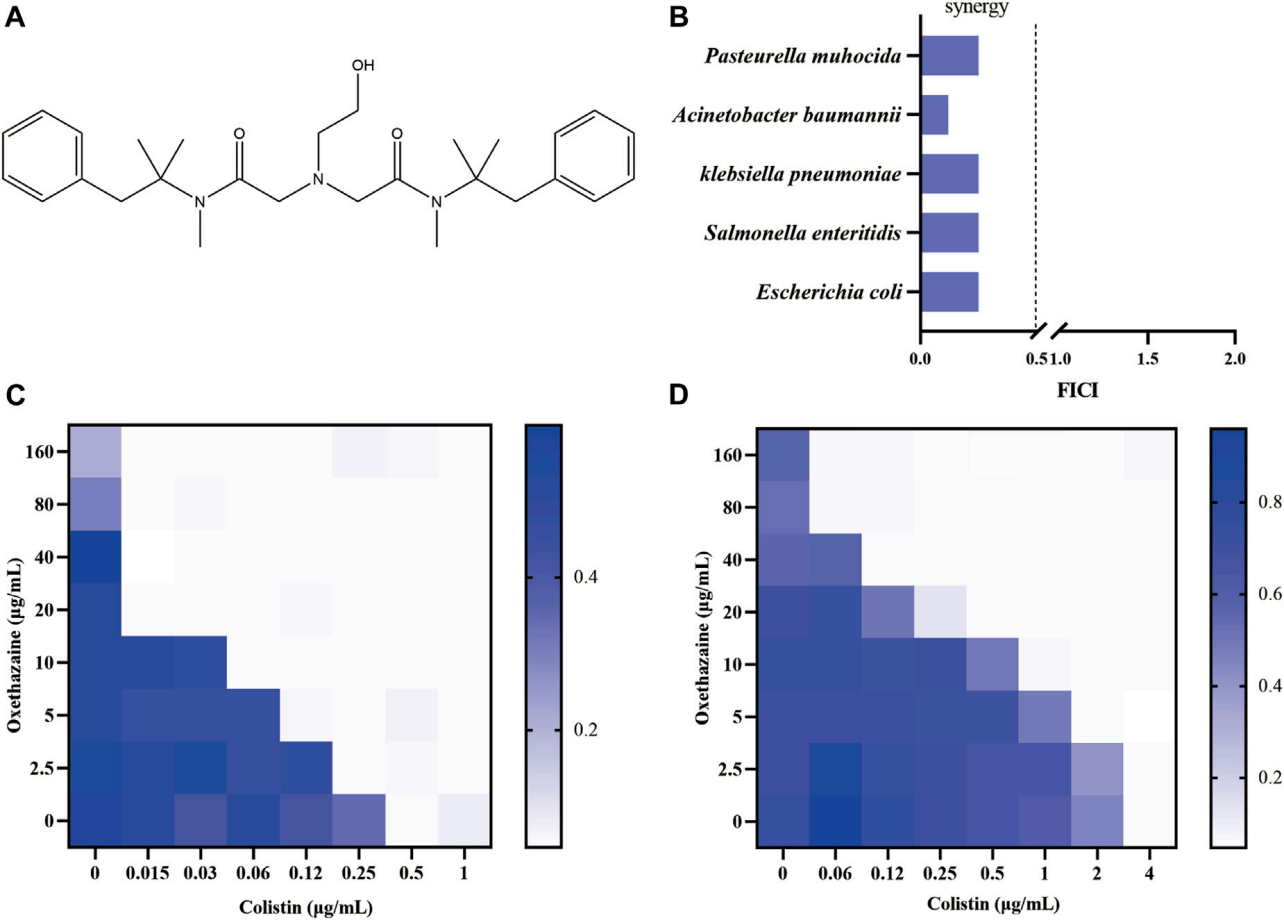
Oxethazaine increased colistin activity against both colistin-resistant and -sensitive *E. coli*. **(A)** Chemical structure of oxethazaine. **(B)** FIC index of oxethazaine combined with colistin against diverse Gram-negative bacteria. Synergy is defined as FICI ≤0.5. **(C)** and **(D)** Chequerboard broth microdilution assays between oxethazaine and colistin against *E. coli* ATCC 25922 and SHP45, respectively. The results of the checkerboard broth microdilution assay were measured by absorbance at 600 nm. Data represent the average of three biological replicates.

Oxethazaine is a stronger local anaesthetic than cocaine and procaine (Seifter et al., 1962). Due to the unique chemical structure (‘double‐anesthetic’) (Figure 1A), oxethazine can maintain function in the low acid environment of pH 1 (Seifter et al., 1962; Bao et al., 2022). Therefore, oxethazaine is frequently used to treat the pain produced by chronic gastritis and duodenal ulcers (Bao et al., 2022). Besides, oxethazaine has also been shown to inhibit proliferation and migration of esophageal cancer cells (Bao et al., 2022). Moreover, oxethazaine has been confirmed to suppress chronic hepatitis B virus replication and capsid assembly (Zhang et al., 2016). However, oxethazaine has rarely been reported in treatment of pathogenic bacteria. Herein, we found that oxethazaine enhanced the activity of colistin against MDR Gram-negative pathogens through various modes of action. This study provides a therapeutic strategy for combating MDR bacteria.

## 2 Methods

### 2.1 Cell culture and drugs

African green monkey kidney cells (Vero) were purchased from the American Type Culture Collection (ATCC) and cultured in Dulbecco’s modified Eagle’s medium (Gibco) supplemented with 10% heat-inactivated FBS (Invitrogen), 1% penicillin–streptomycin and 1% sodium pyruvate (Sigma-Aldrich) at 37°C in a 5% CO_2_ atmosphere. Oxethazaine (CAS no. 126-27-2) and colistin sulfate salt (CAS no. 1264-72-8) were purchased from GlpBio and Sigma-Aldrich, respectively.

### 2.2 Strains

The standard strains for this study were purchased from the China General Microbiological Culture Collection Center (CGMCC), *Escherichia coli* SHP45 and other clinical strains were isolated and preserved by our laboratory. MG1655-Δ*waaC* was previously constructed in our laboratory (Li et al., 2023).

### 2.3 Antimicrobial susceptibility test

The clinical and laboratory standards institute (CLSI 2021) guideline was used to determine the minimum inhibitory concentration (MIC) of oxethazaine and other antibiotics (CLSI, 2021). The combined effect of oxethazaine with other antibiotics was determined using the checkerboard method. Briefly, the single colonies were cultured in Mueller–Hinton broth (Qingdao Haibo Biotechnology Co., Ltd.) and then diluted to 10^6^ CFU/mL. Antibiotics were serial diluted 2-fold in Mueller–Hinton broth, and an equal volume of bacterial suspension (10^6^ CFU/mL) was added in a 96-well microtiter. The MIC values were defined as the lowest antibiotic concentrations with no visible bacterial growth after incubation at 37°C for 18 h. The FICI value is calculated from the MIC value of the drug alone. The specific calculation method of the FICI is as follows:

$$\text{FICI}={\text{FICI}}_{a}+{\text{FICI}}_{b}=\frac{{\text{MIC}}_{\text{ab}}}{{\text{MIC}}_{a}}+\frac{{\text{MIC}}_{\text{ba}}}{{\text{MIC}}_{b}}$$

MIC_a_ is the MIC of A alone, MIC_ab_ is the MIC of compound A in combination with compound B, MIC_b_ is the MIC of compound B alone, and MIC_ba_ is the MIC of compound B in combination with compound A. FICI ≤0.5 was deemed as synergistic (Song et al., 2020).

### 2.4 Safety assay

Sterile sheep red blood cells (RBC) were used to evaluate hemolytic activity as previously described with slight modifications (Liu et al., 2020). In brief, RBC were washed with PBS for three times, then 8% suspension of RBC was treated with colistin (0.12, 0.25, 0.5, 1, 4, 8 and 16 μg/mL) with or without oxethazaine (20 μg/mL). At the same time, PBS and Triton X-100 were used as the negative and positive control, respectively. After 1 h incubation at 37°C, the cells were centrifuged at 3,500 *g* for 8 min, and the absorbance of the supernatant was determined at 576 nm. The haemolysis rate was calculated according to the absorbance of positive control and the blank control. All experiments were carried out at least twice.

The cytotoxicity of Vero cells was detected by cell counting kit-8 (CCK-8) assay (Solarbio life sciences) based on previous report (Zhang et al., 2023). The cells were plated in a 96-well plate at a density of 3 × 10^4^ cells per well and treated with colistin (0.5, 1, 2, 4, 16, 32, 64 and 16 μg/mL) or the combination of colistin (0.5, 1, 2, 4, 16, 32, 64 and 16 μg/mL) and oxethazaine (20 μg/mL). After cultured 4 h at 37°C and 5% CO_2_, 10% CCK-8 solution was added to 100 μL of medium in each well and the culture maintained for an additional 2 h, cells were analyzed with absorbance at 450 nm.

### 2.5 Time-dependent killing assay

An overnight culture of *E. coli* ATCC 25922 and *E coli* SHP45 was diluted 1:1000 in fresh LB broth (Qingdao Haibo Biotechnology Co., Ltd.), and 2 mL bacterial suspension was placed in the 10 mL centrifuge tube. Then the bacteria were treated with 0.5×MIC of colistin, oxethazaine (20 μg/mL) or the combination of colistin and oxethazaine. After incubated at 37°C, 180 rpm for 0, 2, 4, 8, 12, 16, 20 and 24 h, 100 μL of bacterial suspension were collected and ten-fold serially diluted in PBS, and the suspensions were plated on LBA plates. After overnight cultivation at 37°C, bacterial colonies were calculated. Experiments were performed in biological triplicate.

### 2.6 Microscopic observation of LIVE/DEAD staining

A LIVE/DEAD BacLight bacterial viability kit was used to evaluate the synergistic antimicrobial effect of colistin and oxethazaine. An overnight culture of *E. coli* ATCC 25922 was adjusted to OD_600_ of 0.5 and incubated with colistin, oxethazaine or their combination. After 30 min, the bacterial cells were washed with PBS and LIVE/DEAD dye was added. The images of *E. coli* were observed using a confocal fluorescence microscope (Leica, Germany). In addition, the dead cells were observed at excitation wavelength 535 nm and emission wavelength 615 nm with an EnSight multimode plate reader.

### 2.7 Outer membrane integrity assay

The integrity of the OM was evaluated using NPN fluorescent dye (Aladdin, China) (Liu et al., 2020; Song et al., 2020). After incubation overnight at 37°C, the *E. coli* ATCC 25922 were washed three times with 5 mM HEPES (5 mM glucose). After 20 min incubation with the NPN dye, the colistin alone, oxethazaine alone, or their combination was added to the bacterial suspensions. After treatment in the dark at 37°C for 30 min, the fluorescence intensity was determined with the excitation wavelength of 350 nm and the emission wavelength of 420 nm with an EnSight multimode plate reader.

### 2.8 Cytoplasmic membrane permeability assay

Propidium iodide PI (Beyotime, China) was used to evaluated the cytoplasmic membrane permeability (Liu et al., 2020). Briefly, overnight cultures of *E. coli* ATCC 25922 were washed three times with PBS and resuspended with PBS to approximately an OD_600_ nm of 0.5. The bacterial suspensions were cultured with propidium iodide PI at 37°C for 20 min, followed by the addition of colistin (0, 1, 2 and 4 μg/mL) alone or combined with 20 μg/mL oxethazaine. After incubation for 30 min at 37°C, fluorescence intensity was monitored using the EnSight^®^ Multimode Plate reader at an excitation wavelength of 535 nm and an emission wavelength of 615 nm. Experiments were performed in biological triplicate.

### 2.9 Cytoplasmic membrane potential

The fluorescent probe DiSC_3_ (Silvestri and van Saene, 2010) (Aladdin, China) was used to analyze the membrane potential of *E. coli* ATCC 25922 (Song et al., 2021). In brief, bacteria were grown on LB broth to reach the exponential phase, then washed twice with PBS and adjusted to OD_600_ of 0.5. After incubation of the bacterial suspension with DiSC_3_ (Silvestri and van Saene, 2010) (0.5 µM) for 20 min, varying concentrations of colistin (0, 1, 2 and 4 μg/mL) with or without oxethazaine were added, and the fluorescence intensity was determined with the excitation wavelength at 622 nm and emission wavelength at 670 nm with an EnSight multimode plate reader. All tests included biological replicates.

### 2.10 ROS measurement

The levels of ROS in *E. coli* ATCC 25922 were measured using the fluorescence probe 2′,7′-dichlorodihydro-fluorescein diacetate (DCFH-DA) (Beyotime, China) with the excitation wavelength at 488 nm and the emission wavelength at 525 nm (Song et al., 2020). In brief, the bacteria were resuspended in PBS to an OD_600_ of 0.5, then the DCFH-DA probe was added to a final concentration of 10 µM. After the bacteria were incubated at 37°C for 20 min, different concentrations of colistin (0, 1, 2 and 4 μg/mL) in the presence or absence of 20 μg/mL oxethazaine was added and the fluorescence intensity was detected.

### 2.11 Scanning electron microscopy (SEM)

The bacterial morphology was observed by the SEM. Briefly, *E. coli* ATCC 25922 in mid-log phase was washed three times with PBS, and resuspended to 10^7^ CFU. Colistin (0.5×MIC), oxethazaine (20 μg/mL) or their combination (0.5×MIC colistin+20 μg/mL oxethazaine) were added and incubated at 37°C for 4 h. Then samples were washed with PBS and fixed overnight in 2.5% glutaraldehyde solution at 4°C. The bacteria were dehydrated with different gradients of ethanol, and the bacterial morphology was observed by the SEM (Hitachi, Japan) after vacuum drying.

### 2.12 LC–MS/MS analysis

The LC–MS/MS assay was prepared according to described earlier, with minor modifications (Lee et al., 2013). A C18 column (5 μm, 150 mm × 2.0 mm I.D.) was used for chromatography and maintained at 40°C. After the bacteria were treated with 20 μg/mL oxethazaine alone or combined with 1 μg/mL colistin, the accumulation of oxethazaine in *E. coli* was performed on the LCMS-8050 system (Shimadzu, Kyoto, Japan).

### 2.13 *Galleria mellonella* infection model

The synergistic antibacterial effect of oxethazaine and colistin was evaluated in the *Galleria mellonella* larvae infection model. The larvae of *G. mellonella* were randomly divided into four groups (n = 10 per group), and injected with 10 μL of 9.8 × 10^4^ CFUs *E coli* SHP45 suspension. After 1 h, *G. mellonella* were subjected to antibiotic treatment, including 20 mg/kg oxethazaine, 2 mg/kg colistin or the combination of 20 mg/kg oxethazaine and 2 mg/kg colistin. At the same time, PBS was used as the control group. Survival of *G. mellonella* in each group was observed.

### 2.14 Mouse sepsis infection model

6–8 weeks old female BALB/C mice (20 ± 2 g) were used for mouse peritonitis-sepsis model. Mice randomly assigned into five groups (n = 8 per group), mice were acclimated for 7 days before infection in the Laboratory Animal Center of South China Agricultural University. Then mice were infected with 200 μL of 1.8 × 10^8^ CFU *E coli* SHP45 suspension via intraperitoneal injection. After 1 h post infection, mice were treated with PBS, colistin (2 mg/kg), oxethazaine (20 mg/kg), or the combination of 2 mg/kg colistin with 20 mg/kg oxethazaine. The number of survival mice in each group was observed. Once the infected mice died, the liver, spleen, and kidney were removed and analyzed bacterial loading and subjected to histological analysis. After 5 days of experimentation, the remaining animals were euthanized by cervical dislocation according to animal welfare related guidelines. The organs were collected under aseptic conditions, one part of the organs was used for homogenization and other for histological analysis (Valiante et al., 2015; Xie et al., 2020).

### 2.15 Statistical analysis

GraphPad Prism 9.0 software was used for statistical analyses. All data are expressed as a mean ± standard deviation (SD). *p*-values were calculated by one-way ANOVA among multiple groups or *t*-test between two groups. *p < 0.05, **p < 0.01.

## 3 Results

### 3.1 Synergy of oxethazaine with colistin against gram-negative pathogens

Firstly, the potential efficacy of oxethazaine and existing antibiotics was evaluated using checkerboard dilution assays. Interestingly, we observed synergistic activity between oxethazaine and colistin against both colistin-resistant *E. coli* SHP45 and colistin-sensitive ATCC 25922, whereas oxethazaine alone had no antibacterial activity (Figures 1C,D). To evaluate whether the synergistic antibacterial activity was specific, six species of Gram-negative bacteria were used to determine the synergistic effect of oxethazine and colistin. As expected, oxethazaine combined with colistin showed a strong synergistic effect against Gram-negative bacteria (Figure 1B, Supplementary Table S1), suggesting that oxethazaine was a potent antibiotic adjuvant to restore the antibacterial activity of colistin.

### 3.2 Oxethazaine potentiates the activity of colistin *in vitro* without obvious toxicity

**FIGURE 2:**
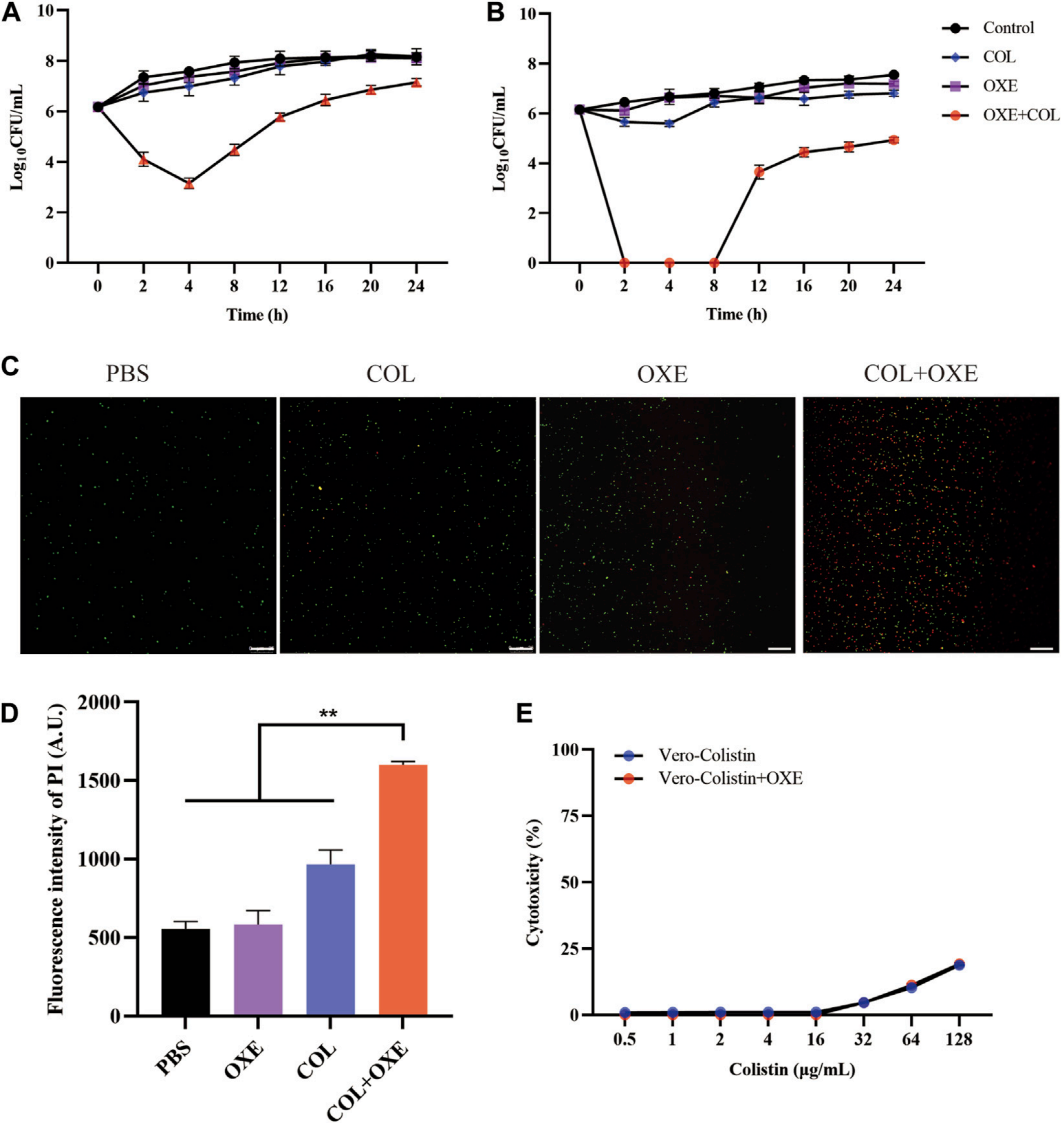
Synergistic antibacterial activity of colistin combined with oxethazaine *in vitro*. Time–kill assays of *mcr*-positive *E. coli* SHP45 **(A)** and *mcr*-negative *E. coli* ATCC 25922 **(B)** was conducted with 0.5×MIC colistin alone, 20 μg/mL oxethazaine alone or their combination (0.5×MIC colistin+20 μg/mL oxethazaine). The number of bacteria per milliliter was measured at different time points within 24 h. **(C)** Fluorescent images of stained bacteria were observed with a confocal fluorescence microscope. *E. coli* ATCC 25922 was treated with 0.5×MIC colistin, 20 μg/mL oxethazaine or the combination of colistin and oxethazaine (0.5×MIC colistin+20 μg/mL oxethazaine). Scar bar, 25 µm. **(D)** The proportion of dead cells was represented by the fluorescence value. The fluorescence intensity was determined with the excitation wavelength of 535 nm and the emission wavelength of 615 nm. **(E)** Cytotoxicity of Vero cells was evaluated by CCK-8. Data are expressed as the mean ± SD of three independent experiments. COL, colistin alone; OXE, oxethazaine alone; COL + OXE, the combination of colistin and oxethazaine.

To further confirm the synergistic antibacterial activity of oxethazaine and colistin, the time-killing experiments were performed on *mcr-*positive or -negative *E. coli*. We found that either 20 μg/mL oxethazaine alone or 0.5×MIC colistin alone showed little bactericidal activity, whereas the combination of oxethazaine and colistin rapidly reduced the number of bacteria with more than 2log_10_ CFU (Figures 2A,B). Furthermore, the synergistic effect was also confirmed by LIVE/DEAD staining, which stains dead bacteria red and viable bacteria green. Compared with colistin or oxethazaine alone, the proportion of dead bacteria increased in the combination group (0.5×MIC colistin+20 μg/mL oxethazaine) (Figures 2C,D).

Toxicity is one of the key factors limiting the clinical application of combination therapy. Therefore, we subsequently evaluated whether the addition of oxethazaine would increase toxicity. Surprisingly, we found no significant increase in hemolytic activity of Sterile sheep red blood cells (RBC) after treatment with the combination of colistin and oxethazaine (Supplementary Figure S1). Moreover, Vero cells were also used to evaluate the cytotoxicity of colistin with or without oxethazaine. Compared to colistin alone, no increased cytotoxicity was observed in the Vero cells after exogenous addition of oxethazaine (Figure 2E). These results indicated that oxethazaine exhibited a robust potentiating effect on the colistin activity without increased toxicity.

### 3.3 Oxethazaine plays a key role in the synergetic effect with colistin

**FIGURE 3:**
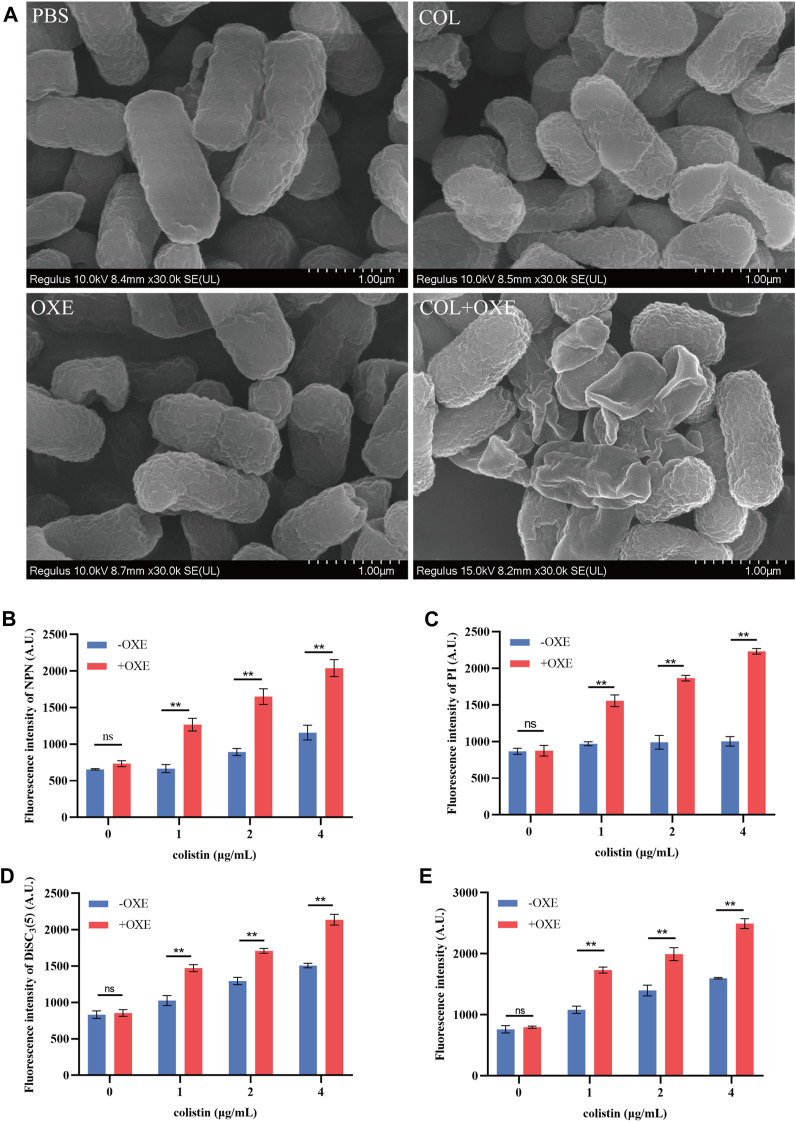
Oxethazaine potentiated the activity of colistin against *E. coli* ATCC 25922. **(A)** Bacterial morphology of *E. coli* ATCC 25922 after exposure to colistin alone, oxethazaine alone or their combination by SEM analysis. Scar bar, 1 µm. **(B)** The outer membrane permeability was measured by the fluorescence intensity of 1-N-phenylnaphthylamine (NPN) after treated with the different concentrations of colistin alone or combined with 20 μg/mL oxethazaine. Fluorescence was measured with the excitation/emission wavelength at 350 nm/420 nm. **(C)** The cytoplasmic membrane permeability was evaluated under the different concentrations of colistin with or without 20 μg/mL oxethazaine. Fluorescence dye propidium iodide (PI) was used to determined the permeability of cytoplasmic membrane. **(D)** The membrane potential of colistin with or without 20 μg/mL oxethazaine was evaluated by the fluorescence dye DiSC3(5). **(E)** The effect of colistin on the ROS accumulation in *E. coli* in the absence or presence of 20 μg/mL oxethazaine. All data are presented as mean ± SD and the significances were determined by nonparametric one-way ANOVA (*p < 0.05, **p < 0.01). OXE, oxethazaine.

**FIGURE 4:**
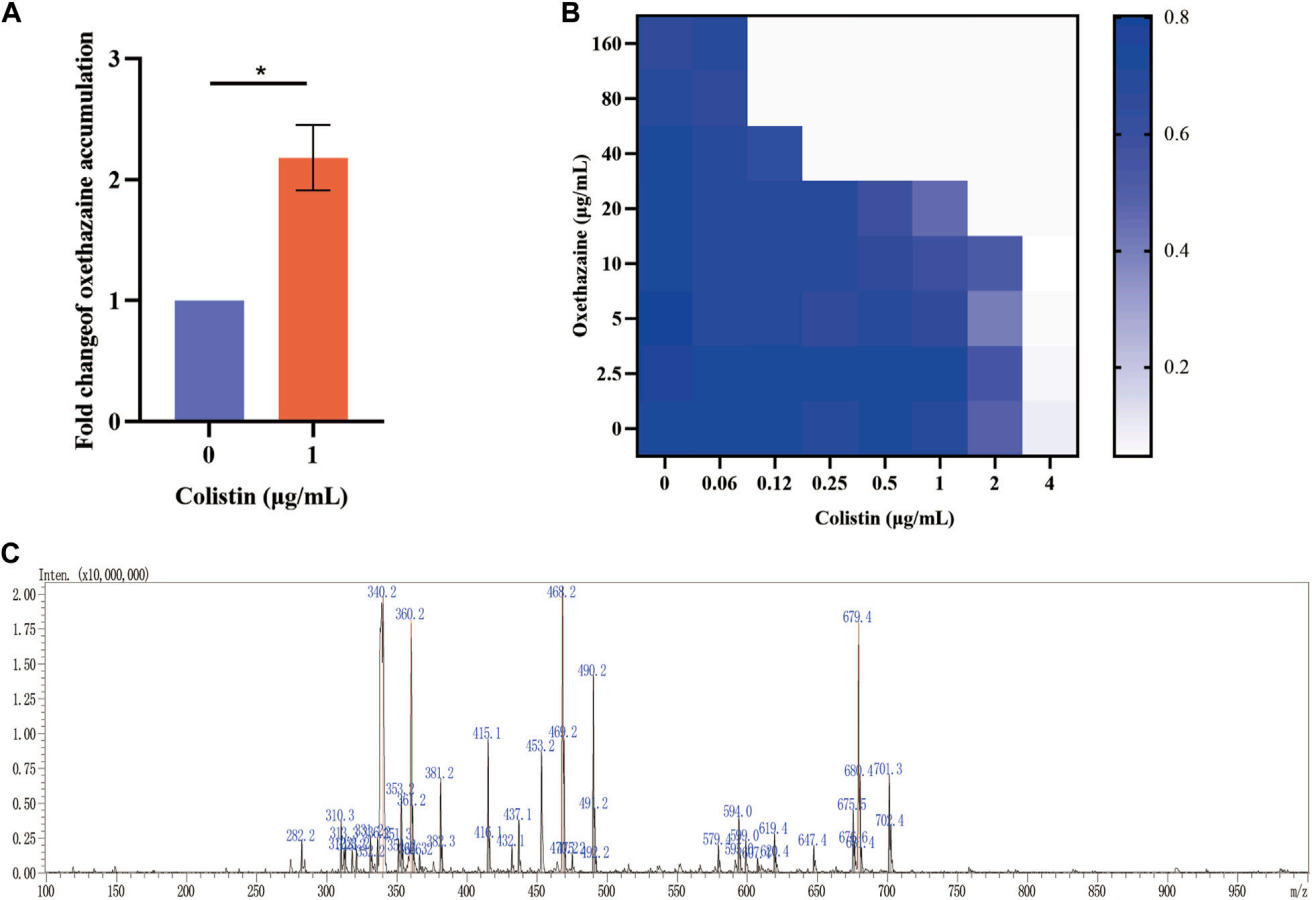
Oxethazaine plays a key role in the synergetic effect with colistin. **(A)** The accumulation of oxethazaine was conducted in the presence or absence of colistin. **(B)** Effects of NAC (6 mmol/L) on the synergistic effect of oxethazaine against Gram-negetive bacteria. **(C)** The MS spectrum of oxethazaine in positive ion mode. All date were presented as mean ± SD. *p* values were calculated with an unpaired, two-tailed Student’s t-test. *p < 0.05.

Bacterial structural morphology is critical for bacterial viability (Srivastava et al., 2020), thus the morphological changes was evaluated by scanning electron microscopy (SEM) analysis. After treatment with either oxethazaine or colistin alone, a slight morphological change was observed. In comparison, the cell surface appeared strongly sunken and shrinkage after treated with the oxethazaine combined with colistin (Figure 3A). Therefore, we hypothesized that the combination might have an effect on the bacterial membranes. To confirm this hypothesis, we first evaluated the effect on the OM using the fluorescent dye N-phenyl-1-naphthylamine (NPN) (Song et al., 2020). As expected, the fluorescence value of colistin was significantly increased in the presence of oxethazaine (*p* < 0.05), whereas only subtle changes in fluorescence intensity was detected after treated with oxethazaine alone (Figure 3B) (*p* > 0.05). In addition, oxethazaine restored antibacterial activity against LPS-deleted strain MG1655-Δ*waaC* (Table 1), indicating that the OM acts as a permeability barrier to prevent oxethazaine from entering the cell. Thus, the intracellular accumulation of agents was detected by LC-MS/MS (Figure 4C, Supplementary Table S2), and we found that colistin significantly promoted the accumulation of oxethazaine (Figure 4A) (*p* < 0.05). Moreover, cytoplasmic membrane (CM) integrity and membrane potential was significantly dissipated in the combination group (Figures 3C,D) (*p* < 0.05).

**TABLE 1:** The MIC of oxethazaine against *E. coli* bacteria.

Strain	Colistin (μg/mL)	Oxethazaine (μg/mL)
MG1655	0.5	>256
MG1655-Δ*waaC*	0.12	5

**FIGURE 5:**
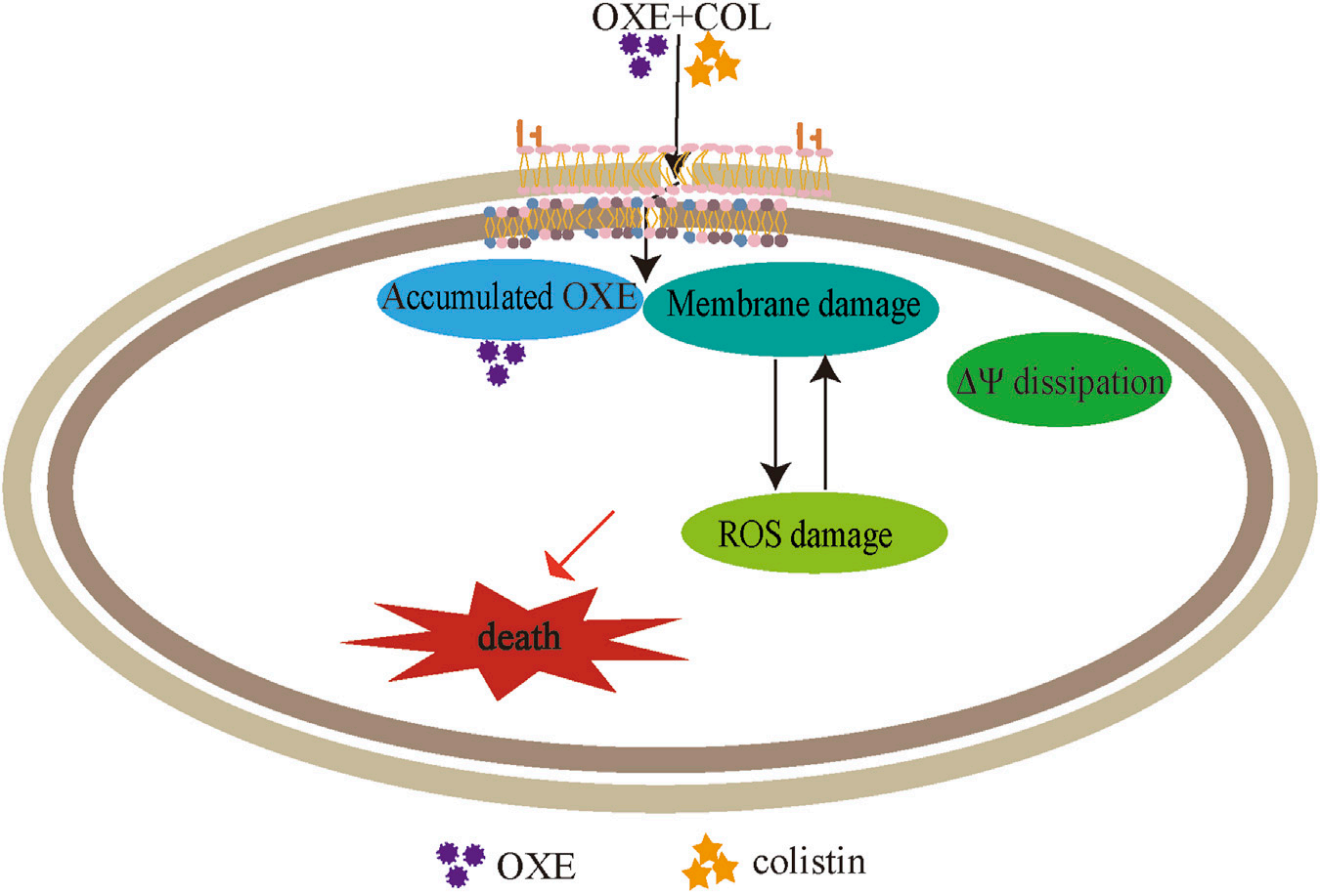
The synergistic mechanism of oxethazaine combined with colistin against MDR pathogens. Oxethazine improves the ability of colistin to destruct bacterial outer membrane and cytoplasmic membrane permeability. At the moment, the combination of colistin and oxethazaine dissipates the membrane potential of cytoplasmic membrane and promotes the accumulation of oxethazaine. In addition, their combination triggers the accumulation of ROS, causing additional damage to the membrane structure resulting in cell death. OXE, oxethazaine.

The integrity of the membrane structure is critical for several bacterial biosynthetic processes (Wang et al., 2022). Notably, we observed that the accumulation of bacterial reactive oxygen species (ROS) was enhanced in a dose-dependent manner after the addition of oxethazaine (Figure 3E). Moreover, exogenous addition of N-acetylcysteine (NAC, 6 mmol/L) can reduce the synergistic effect of oxethazaine combined with colistin (Figure 4B), revealing that ROS play a critical role in their synergistic process. These results indicated that oxethazaine enhanced the antibacterial activity of colistin by multiple modes of action (Figure 5).

### 3.4 The efficacy of the combination *in vivo*

**FIGURE 6:**
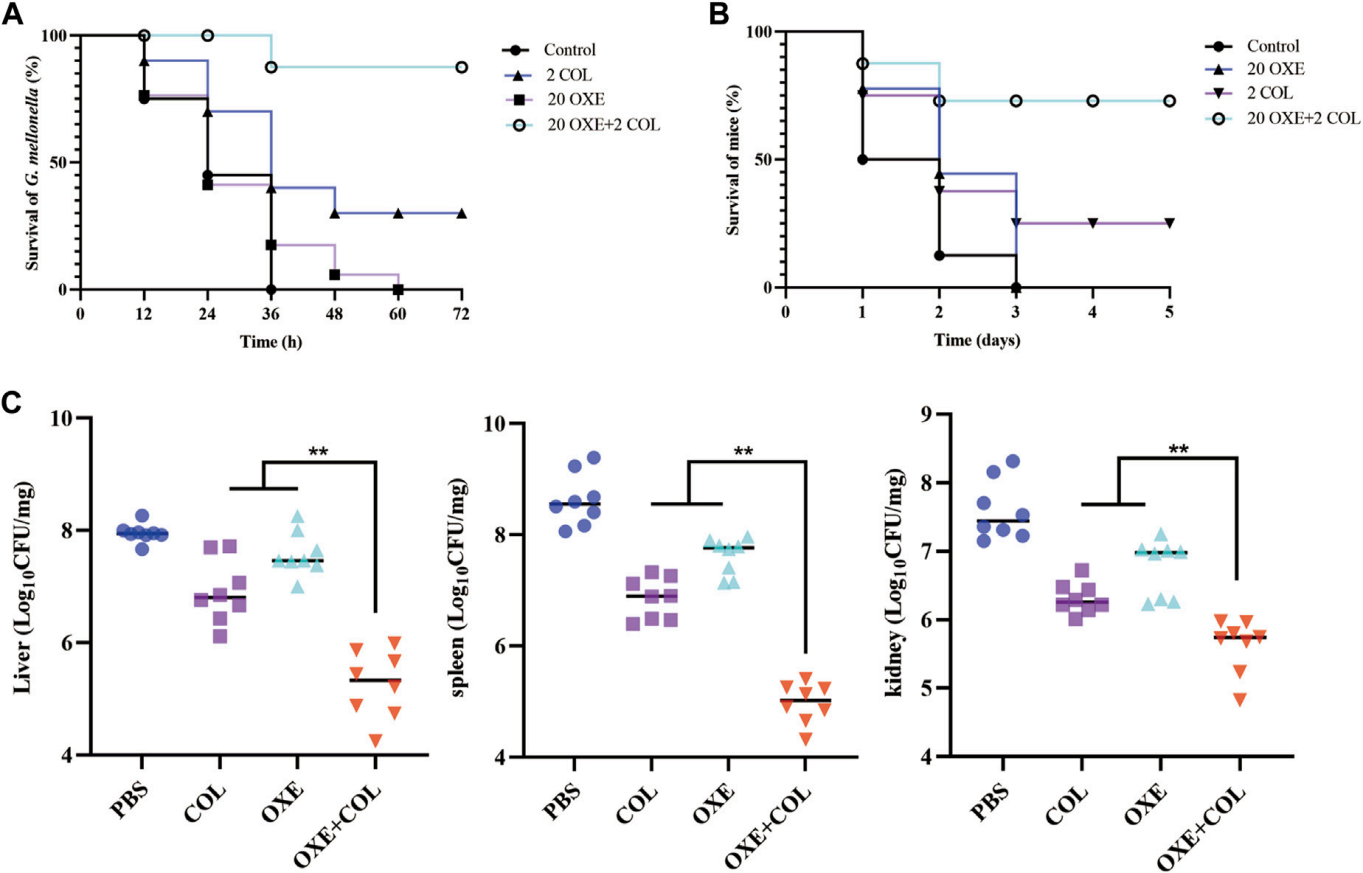
Oxethazaine rescued the therapeutic effect of colistin in animal infection models. **(A)** Survival rates of G. mellonella larva (n = 10 per group) treated with 20 mg/kg oxethazaine alone, 2 mg/kg colistin alone or their combination (20 mg/kg oxethazaine +2 mg/kg colistin). **(B)** Survival rates of peritonitis-sepsis mice (n = 8 per group) infected with 1.8 × 108 CFU *E. coli* SHP45. The combination therapy of oxethazaine and colistin (20 mg/kg+2 mg/kg) improved the Survival rates of mice. **(C)** Bacterial loads in different organs (liver, spleen and kidney) were counted in mouse infection model. *p* values were calculated by nonparametric one-way ANOVA (*p < 0.05, **p < 0.01). COL, 2 mg/kg colistin alone; OXE, 20 mg/kg oxethazaine alone; COL + OXE, the combination of 2 mg/kg colistin and 20 mg/kg oxethazaine.

Given the strong potentiation of oxethazaine on colistin *in vitro*, we further assessed whether their combination had potential efficacy *in vivo* infected with *E. coli* SHP45 using the *G. mellonella* model and the mouse peritonitis/sepsis model. As expected, the survival rate of infected larvae was increased after treatment with the combination of oxethazaine and colistin (20 mg/kg+2 mg/kg), whereas the monotreatment only displayed a slight therapeutic effect (Figure 6A). Accordingly, the significant therapeutic efficacy was also confirmed in the mouse model. 75.0% of the mice were protected in the therapy of 20 mg/kg oxethazaine combined with 2 mg/kg colistin. In contrast, all mice in the PBS and oxethazaine monotherapy groups died within 3 days (Figure 6B). Besides, the pathological changes in each organ were alleviated accordingly (Figure 6C). Moreover, compared with the monotherapy groups, combinational therapy significantly reduced the bacterial burden in various organs of mice (Supplementary Figure S2). These findings revealed that oxethazaine effectively rescued colistin activity to combat the MDR *E. coli in vivo*.

## 4 Discussion

The dissemination of resistant pathogens far outpaces development rate of new antibiotics. Furthermore, there are few antibiotics against Gram-negative bacteria in the drug discovery pipeline (Li et al., 2014). Therefore, there is an urgent need to develop antibiotic adjuvant strategies to substantially fill the vacancy of new antibiotics. Antimicrobial adjuvants not only extend the life of existing antibiotics but also broaden the antimicrobial spectrum (Allen and Brown, 2019; Liu et al., 2021). The classic example is the antibiotic adjuvant β–lactamase inhibitor clavulanic acid, which has a potentiating effect on β-lactam antibiotic therapy (Worthington and Melander, 2013). Given the global prevalence of plasmid-mediated acquired colistin resistance (Wang et al., 2018), the discovery of effective colistin adjuvants is critical.

Currently, a variety of colistin adjuvants have been discovered, including plant-derived (Wang et al., 2018; Song et al., 2021), chemically synthesized (Kathayat et al., 2020; Li et al., 2023), and FDA-approved (Stokes et al., 2017; Torres et al., 2018). Among these, the discovery of new uses for FDA approved old drugs is cheaper and faster than the discovery of new drugs from scratch (Boucher et al., 2013; Farha and Brown, 2019). Herein, we screened drugs that had a synergistic effect when combined with colistin from FDA-approved drugs, providing multiple strategies for the antibiotic selection pipeline. Fortunately, we found that oxethazaine, as a potential antibiotic adjuvant, could effectively restore the antibacterial activity of colistin against MDR *E. coli*. The time-killing assay confirmed further that the colistin combined with oxethazaine inhibited bacterial growth within 8 h compared with colistin alone. However, the observed bacterial regrowth was observed at later stages may be due to the half-life of colistin is 4 h (Bergen et al., 2010). In addition, the combination of oxethazaine and colistin showed a synergistic effect against all strains tested, which is consistent with previous report (Hind et al., 2019). However, the specific synergistic antibacterial effect in the combination of colistin and oxethazaine may be related to difference in the strains.

In the clinic, toxicity is an important evaluation index of drugs. Clinical therapy of colistin has historically been limited due to severe side effects (Spapen et al., 2011; Nang et al., 2021). As we all know, colistin was first discovered in the 1940s (Lim et al., 2010), but it was replaced by other antibiotics in the 1970s due to its nephrotoxicity (Wolinsky and Hines, 1962; Osei Sekyere et al., 2020). The most important significance of the discovery of colistin adjuvants is that they can increase the antibacterial activity of colistin, reducing the clinical dosage of colistin to be used in clinical treatment, thereby greatly reducing its toxicity and side effects. Notably, oxethazaine, an over-the-counter, has wide safety margins when administered orally, subcutaneously, intramuscularly, and rectally (Prado et al., 2017; Bao et al., 2022). When combined with colistin, oxethazaine had a negligible effect on colistin toxicity *in vitro*.

Bacterial membranes are a valuable target for bacterial eradication, especially persistent bacteria (Cui et al., 2016). Interestingly, we found that oxethazaine could increase the membrane damage of colistin (Figure 3B), which is similar to that of equisetin to enhance the activity of colistin (Zhang et al., 2021). Endogenous ROS are crucial for bactericidal agents and increased ROS can make bacteria more susceptible to antibiotics (Brynildsen et al., 2013; Van Acker and Coenye, 2017). Furthermore, the accumulation of ROS can aggravate membrane damage and further promote bacterial death (Rowe-Magnus et al., 2019). High levels of ROS can induce oxidative stress, resulting in the damage to lipids, DNA and cytoplasmic proteins (Yuan et al., 2014; Rowe-Magnus et al., 2019). More importantly, the accumulation of ROS further exacerbated membrane damage leading to bacterial death. The permeability barrier of the OM is an important reason why many drugs are ineffective against Gram-negative bacteria (Nikaido, 2003; May and Grabowicz, 2018; Theuretzbacher et al., 2020). Oxethazaine alone has no antibacterial activity against *E. coli*, but it has synergistic antibacterial activity against LPS deletion strains MG1655-Δ*waaC*, indicating that OM might be a potential barrier preventing oxethazaine from reaching intracellular targets. Notably, this combination therapy remains effective in strains that are resistant to colistin due to chromosomal mutations (Lima et al., 2017; MacNair et al., 2018). Nevertheless, more in-depth synergistic mechanisms need to be studied *in vitro*. More importantly, the efficacy of combination therapy *in vivo* is crucial. Considering the high safety of oxethazaine (Prado et al., 2017; Bao et al., 2022), we believe it is an attractive lead compound as an adjuvant to colistin. However, more animal experiments should be carried out for clinical application in the future.

## 5 Conclusion

In this study, we found that FDA-approved oxethazaine restored the antibacterial activity of colistin against Gram-negative bacteria through multiple strategies, including enhancing the ability of colistin to disrupt OM and CM, promoting intracellular ROS accumulation and intracellular oxethazaine accumulation to exert antibacterial activity. Further, synergistic therapy of oxethazaine combined with colistin protected mice from *mcr*-positive *E*. *coli* bacterial infection *in vivo*. Oxethazaine is a potential candidate antimicrobial adjuvant candidate for colistin, providing an alternative strategy for the treatment of infections caused by MDR Gram-negative pathogens.

## Data Availability

The original contributions presented in the study are included in the article/Supplementary Material, further inquiries can be directed to the corresponding author.
