# Supplementary material for: The synergistic antibacterial activity and mechanism of colistin-oxethazaine combination against gram-negative pathogens
Source: Front Pharmacol. 2024 Mar 21;15:1363441. doi: 10.3389/fphar.2024.1363441 (PMC10991713; doi:10.3389/fphar.2024.1363441)
Supplement: Supplementary file 1 [file DataSheet1.DOCX]

**The synergistic antibacterial activity and mechanism of colistin-oxethazaine combination against Gram-negative pathogens**

Jie Li ^a, b, c^, Ning Han ^a, b, c^, Yangyang Li ^a, b, c^, Feifei Zhao ^a, b, c^, Wenguang Xiong ^a, b, c^, Zhenling Zeng ^a, b, c^ *

a Guangdong Provincial Key Laboratory of Veterinary Pharmaceutics Development and Safety Evaluation, College of Veterinary Medicine, South China Agricultural University, Guangzhou 510642, China.

b National Laboratory of Safety Evaluation (Environmental Assessment) of Veterinary Drugs, South China Agricultural University, Guangzhou 510642, China.

c National Risk Assessment Laboratory for Antimicrobial Resistance of Animal Original Bacteria, South China Agricultural University, Guangzhou 510642, China.

*Corresponding author

E-mail: zlzeng@scau.edu.cn

**
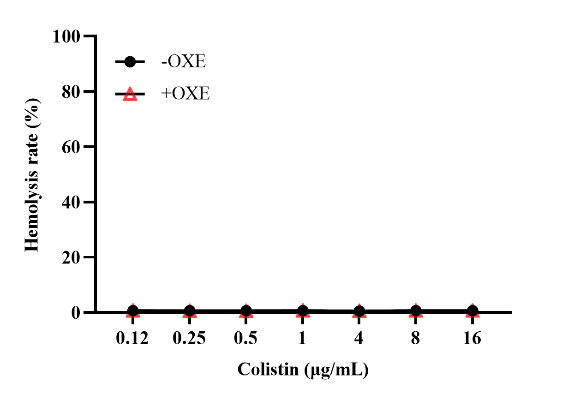
**

**Figure S1** Hemolytic activity of colistin alone or combined with oxethazaine on sheep red blood cells. OXE, oxethazaine alone


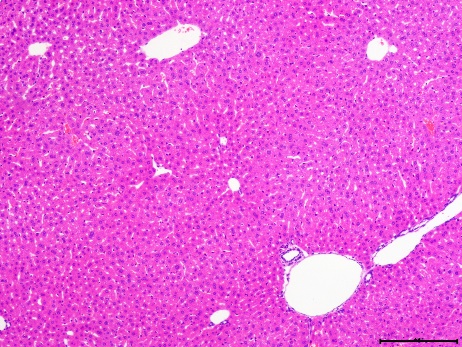

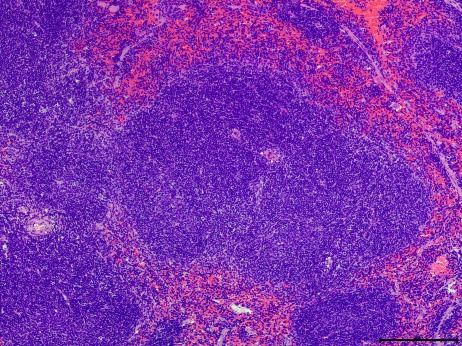

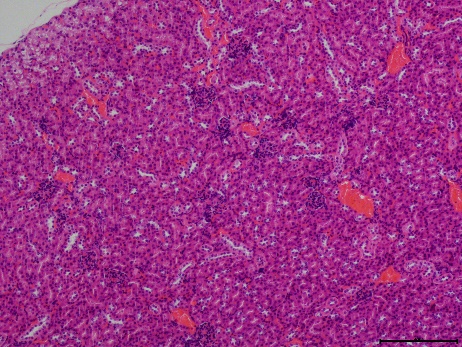


control

liver

spleen

kidney

OXE

COL

OXE+COL


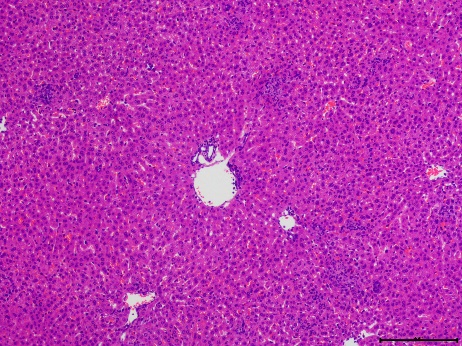

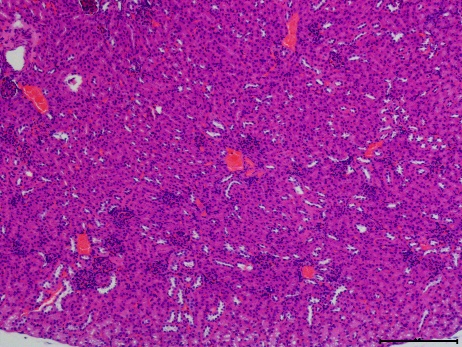

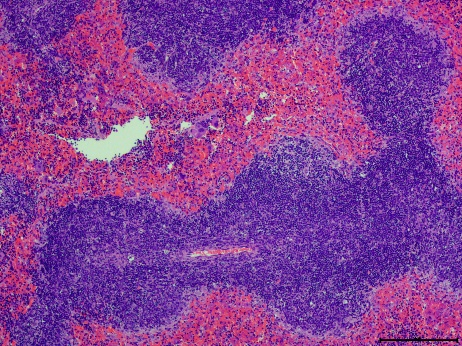

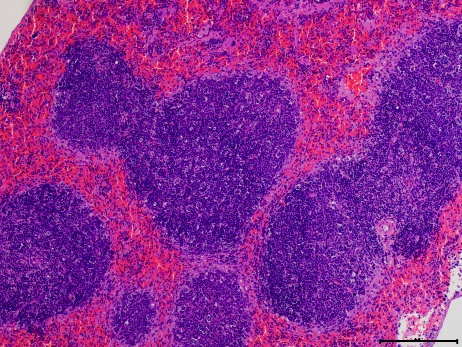

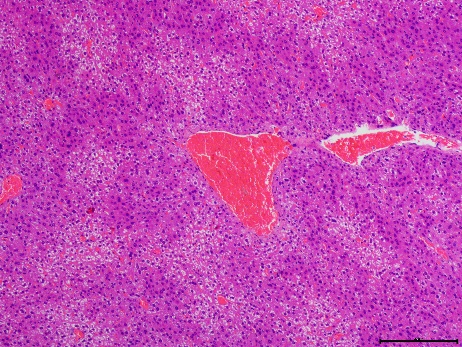

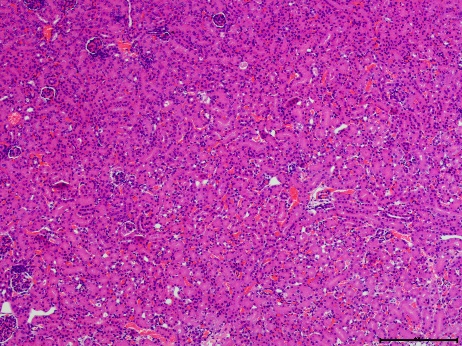

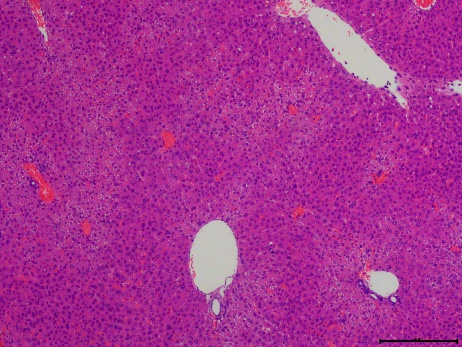

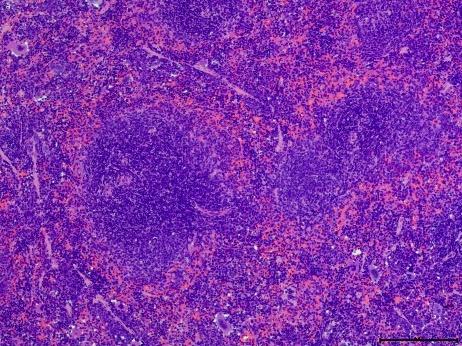

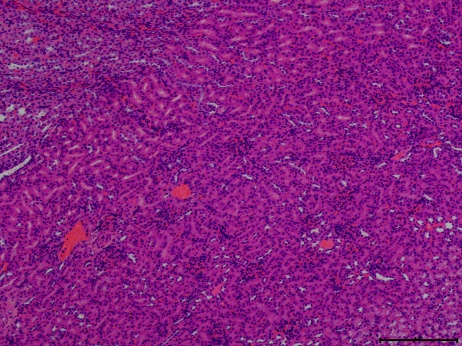


**Figure S2** Histologic analysis of mouse organs using hematoxylin-eosin (HE) staining.

The liver, spleen, and kidney were histological analysis. The results were representative of three biological repeats. Scar bar, 200 μm. COL, colistin alone; OXE, oxethazaine alone; COL+OXE, the combination of colistin and oxethazaine.

**Table S1** All strains used in this study

| strains | *mcr-1* | Source/Reference | strains | *mcr-1* | Source/Reference |
| --- | --- | --- | --- | --- | --- |
| *Escherichia coli* | reference strain | ATCC 25922 | *S. typhimurium* 002S | - | In this study |
| *E. coli* SHP45 | *+* | (1) | *S. typhimurium* 19134 | - | In this study |
| *E. coli* GDQ20D48 | *+* | In this study | *k. pneumoniae*  K22-2 | - | In this study |
| *E. coli* GDH21D8 | *+* | In this study | *k. pneumoniae*  K195 | - | In this study |
| *E. coli* GDH21D35 | - | In this study | *klebsiella pneumoniae* | reference strain | ATCC 700603 |
| *E. coli* GDQ8D105 | *+* | (2) | *A. baumannii* 131174 | - | In this study |
| *E. coli* GDQ8P37 | *+* | (2) | *Acinetobacter. baumannii* | reference strain | ATCC 19606 |
| *Salmonella typhimurium* | reference strain | ATCC 14028 | *A. baumannii* 131398 | - | In this study |
| *S. typhimurium* 26FS14 | *+* | (3) | *A. baumannii* 130939 | - | (2) |
| *S. typhimurium* S226 | *+* | (3) | *P*. *muhocida* 117 | - | (2) |
| *S. typhimurium* S235 | *+* | (3) | *Pasteurella muhocida* | reference strain | CVCC 434 |
| *S. typhimurium* 20S | - | In this study | *P. muhocida* 202 | - | In this study |
| *S. typhimurium* F19062S | - | (2) | *P. muhocida* 23 | - | (2) |

ATCC, American Type Culture Collection; CVCC, China Veterinary Culture Collection Center.

**Table S2** MRM parameters for the determination of oxethazaine by LC-MS/MS

| **Compound** | **Precursorion（m/z）** | **Product ions（m/z）** | **Retention**  **Time (ms)** | **Collision energy (eV)** |
| --- | --- | --- | --- | --- |
| oxethazaine | 468.2 | 74.1  145.2* | 50  50 | -44  -27 |

**Reference**

1. Liu YY, Wang Y, Walsh TR, Yi LX, Zhang R, Spencer J, Doi Y, Tian GB, Dong BL, Huang XH, Yu LF, Gu DX, Ren HW, Chen XJ, Lv LC, He DD, Zhou HW, Liang ZS, Liu JH, Shen JZ. 2016. Emergence of plasmid-mediated colistin resistance mechanism MCR-1 in animals and human beings in China: a microbiological and molecular biological study. Lancet Infectious Diseases 16:161-168.

2. Li J, Zhang X, Han N, Wan P, Zhao F, Xu T, Peng X, Xiong W, Zeng Z. 2023. Mechanism of Action of Isopropoxy Benzene Guanidine against Multidrug-Resistant Pathogens. Microbiology Spectrum 11:e0346922.

3. Kong L, Lu Y, Yang L, Zhang W, Zuo B, Peng X, Qin Z, Li M, Zeng Z, Zeng D. 2022. Pharmacokinetics and Pharmacodynamics of Colistin Combined With Isopropoxy Benzene Guanidine Against mcr-1-Positive Salmonella in an Intestinal Infection Model. Frontiers In Microbiology 13:907116.
